# Comparative clinical effectiveness and cost effectiveness of endovascular strategy *v* open repair for ruptured abdominal aortic aneurysm: three year results of the IMPROVE randomised trial

**DOI:** 10.1136/bmj.j4859

**Published:** 2017-11-14

**Authors:** IMPROVE Trial Investigators

## Abstract

**Objective** To assess the three year clinical outcomes and cost effectiveness of a strategy of endovascular repair (if aortic morphology is suitable, open repair if not) versus open repair for patients with suspected ruptured abdominal aortic aneurysm.

**Design** Randomised controlled trial.

**Setting** 30 vascular centres (29 in UK, one in Canada), 2009-16.

**Participants** 613 eligible patients (480 men) with a clinical diagnosis of ruptured aneurysm, of whom 502 underwent emergency repair for rupture.

**Interventions** 316 patients were randomised to an endovascular strategy (275 with confirmed rupture) and 297 to open repair (261 with confirmed rupture).

**Main outcome measures** Mortality, with reinterventions after aneurysm repair, quality of life, and hospital costs to three years as secondary measures.

**Results** The maximum follow-up for mortality was 7.1 years, with two patients in each group lost to follow-up by three years. After similar mortality by 90 days, in the mid-term (three months to three years) there were fewer deaths in the endovascular than the open repair group (hazard ratio 0.57, 95% confidence interval 0.36 to 0.90), leading to lower mortality at three years (48% *v* 56%), but by seven years mortality was about 60% in each group (hazard ratio 0.92, 0.75 to 1.13). Results for the 502 patients with repaired ruptures were more pronounced: three year mortality was lower in the endovascular strategy group (42% *v* 54%; odds ratio 0.62, 0.43 to 0.88), but after seven years there was no clear difference between the groups (hazard ratio 0.86, 0.68 to 1.08). Reintervention rates up to three years were not significantly different between the randomised groups (hazard ratio 1.02, 0.79 to 1.32); the initial rapid rate of reinterventions was followed by a much slower mid-term reintervention rate in both groups. The early higher average quality of life in the endovascular strategy versus open repair group, coupled with the lower mortality at three years, led to a gain in average quality adjusted life years (QALYs) at three years of 0.17 (95% confidence interval 0.00 to 0.33). The endovascular strategy group spent fewer days in hospital and had lower average costs of −£2605 (95% confidence interval −£5966 to £702) (about €2813; $3439). The probability that the endovascular strategy is cost effective was >90% at all levels of willingness to pay for a QALY gain.

**Conclusions** At three years, compared with open repair, an endovascular strategy for suspected ruptured abdominal aortic aneurysm was associated with a survival advantage, a gain in QALYs, similar levels of reintervention, and reduced costs, and this strategy was cost effective. These findings support the increasing use of an endovascular strategy, with wider availability of emergency endovascular repair.

**Trial registration** Current Controlled Trials ISRCTN48334791; ClinicalTrials NCT00746122.

## Introduction

Ruptured abdominal aortic aneurysm remains a common vascular emergency with high mortality rates. There have been three recent European randomised trials of endovascular versus open repair for ruptured aneurysm, including the IMPROVE trial. None of the individual trials or their combined data showed a significant survival benefit during the acute period (0-90 days) with endovascular repair.^1^ ^2^ ^3^ This challenges the data from systematic reviews of observational studies, which show a much lower operative mortality after endovascular aneurysm repair (EVAR).^4^ ^5^ Few recent comparative studies have followed patients undergoing either EVAR or open repair for ruptured abdominal aortic aneurysm in the mid or longer term (beyond a year after rupture). Studies, including one from the Vascular Study Group of New England,^6^ one from the Amsterdam cohort with ruptured aneurysm,^7^ and a comparison of an endovascular first strategy with an open repair first strategy in Sweden,^8^ were mainly non-randomised and retrospective and could be confounded by aortic morphology^9^ and other unmeasured factors. Such data have suggested that, after three to five years, survival was similar for those having endovascular and open repair and that patients’ comorbidities and shock on admission were the main determinants of longer term survival.^6^ The Amsterdam cohort study, which was dominated by open repair patients, showed that any early survival benefit of EVAR had been eroded by two years, and thereafter survival was similar in patients treated by open or endovascular repair, with about 50% of patients remaining alive at three years.^7^ The Amsterdam study also showed that, for those discharged alive, later reinterventions were more common after endovascular than open repair. Therefore, the mid-term clinical and cost effectiveness of EVAR or an endovascular strategy for the management of ruptured aneurysm remains uncertain.

There has been considerable reorganisation of vascular services to provide higher volume centres for elective surgery in the UK and elsewhere.^10^ Further changes might be necessary to optimise the use of scarce resources, including intensive care, and to ensure equitable access to complex emergency surgery.^11^ The logistics of providing an endovascular service for ruptured abdominal aortic aneurysm are considerable with regard to the availability of appropriate staff, facilities, and consumables, and many centres in the IMPROVE trial could not offer this service every day of the week. Better evidence is required to stimulate organisational change, particularly further evidence of the effect of an endovascular strategy on mortality, reintervention rates, health related quality of life (QoL), and cost beyond one year follow-up. We investigated the hypothesis that in the mid-term (by three years) an endovascular strategy remains both clinically effective and cost effective.

## Methods

### Design

IMPROVE (ISRCTN 48334791) was a multicentre trial of unselected patients aged over 50 in whom a senior hospital clinician had made a clinical diagnosis of ruptured aortic aneurysm. Patients were randomised to either an endovascular strategy (immediate computed tomography and emergency EVAR if morphologically feasible) or emergency open repair. Patients were usually randomised in the emergency room, before computed tomography and anaesthesiology opinion. Therefore, open repair was the specified treatment for patients who were morphologically unsuitable for EVAR in the endovascular strategy group. In the open repair group, computed tomography was not compulsory but was used in 90% of patients. The trial methods, 30 day, and one year outcomes have been published elsewhere.^2^ ^12^ Soon after the completion of recruitment in 2013, with the observation that about 50% patients remained alive at three years, the trial was extended to provide three year outcomes for all patients. The same outcomes collected at one year were collected at three years, on a post hoc basis, as three year outcomes were not originally registered at the start of the trial because further funding for longer term follow-up could be sought only once all patients had been recruited. The study protocol was updated in August 2013 to include the three year outcomes: this, and the statistical analysis plans, are available from the trial websites (www.improvetrial.org and www.imperial.ac.uk/medicine/improvetrial).

### Centres, randomisation, and patients

This trial was conducted in 29 British and one Canadian centres with proved competence in emergency EVAR. An independent contractor provided central telephone computer generated randomisation (1:1), stratified by centre with variable block size, which automatically provided date and time of randomisation. There was no blinding. The study randomised 613 patients from September 2009 to July 2013 and followed them up to July 2016. The trial guidelines for suitability for EVAR were aneurysm neck diameter ≤32 mm, aneurysm neck length ≥10 mm, and neck angulation <60°.^13^ As the radiological diagnosis of rupture can be difficult,^14^ experts in a core laboratory based at St George’s Hospital, London, later reviewed computed tomograms.

### Outcomes

The primary outcome was total mortality, with secondary outcomes at three years including reinterventions related to the aneurysm, QoL, resource use, costs, quality adjusted life years (QALYs), and incremental cost effectiveness. Total mortality in the UK was from data linkage with the Office for National Statistics (ONS) and locally in Canada. Trained local coordinators were responsible for the collection of prospective data on resource use including readmissions and reinterventions related to the aneurysm and its repair and QoL data using EuroQol questionnaire (five dimension, three level version; EQ-5D) for all patients undergoing aneurysm repair. Related reinterventions were categorised as arterial, related to laparotomy, or other and classified according to whether they were for a life threatening condition or not (table A in appendix 1). The completeness of reintervention and readmission data was verified by detailed audit in Scotland and Canada and additionally cross checked against an administrative dataset (hospital episode statistics) for reinterventions in England, including those at non-trial hospitals (the source of data used for analyses is shown in table B in appendix 1). The main metric of cost effectiveness was the incremental net monetary benefits, which is calculated by valuing incremental QALYs at a recommended threshold of £30 000 (€33 650, $39 540) per QALY, and then subtracting the incremental costs.^15^ The endovascular strategy would be judged relatively cost effective if the estimated incremental net monetary benefit was positive at this threshold, but we also considered a range of alternative thresholds.

### Patient involvement

Patients who had survived an earlier repair of ruptured aneurysm and their families were involved in the design of the trial and choice of outcomes (particularly the two stage ethical approval, reporting of place of discharge from hospital, and adverse reinterventions). The wife of a previous patient was included in the trial steering committee to oversee the conduct of the trial. Patients were not involved in the recruitment process. Patients’ quality of life was assessed at three time points. A short animated video about the trial and its results is available for patients and the public at www.improvetrial.org and will be made available to the Circulation Foundation.

### Statistical analysis

Analyses of the full trial cohort were performed on an intention to treat basis. Mortality was assessed with standard survival analysis techniques, including Kaplan-Meier curves and Cox proportional hazard models, with all available follow-up and additionally for the time periods 0-three months (acute) and three months to three years (mid-term). Primary analyses were unadjusted for baseline variables with secondary analyses adjusted for sex, age (continuous measure), Hardman index^16^ (morbidity score), lowest systolic blood pressure, and aneurysm neck length (when appropriate). We used multiple imputation with chained equations to account for missing data for baseline covariates, resource use, costs, and the EQ-5D utility score (further details are in table C in appendix 1.^17^ All adjustment variables except age were also used for subgroup analysis but, given the multiple tests performed, an interaction test P value of <0.01 was required to claim strong evidence of differences between subgroups. The prespecified analysis plan also identified a principal sensitivity analysis restricted to the 502 patients with a confirmed diagnosis of rupture in whom repair was started. In this analysis data were analysed according to the group assigned at randomisation. We compared the proportion surviving at three years after randomisation between the randomised groups using a Pearson’s χ^2^ test without continuity correction and reported odds ratios using logistic regression. Hazard ratios corresponding to time to first reintervention related to the aneurysm and time to any reinterventions related to the aneurysm and its repair were obtained from Cox regression models, the latter with a multiple failure time model.^18^

We calculated the EQ-5D utility index score by combining the EQ-5D health profile of each patient with health state preference values from the UK general population^19^ and compared the resultant mean QoL utility scores with unpaired *t* tests. For patients discharged without aneurysm repair, quality of life was estimated as previously^12^ (further details in appendix 2). QALYs up to three years were calculated by valuing each patient’s survival time by their QoL at three, 12, and 36 months according to the “area under the curve” method.^20^ Detailed resource use and costs within three years of randomisation were measured in accordance with international guidelines^21^ and reported from a hospital and personal social services perspective as recommended by the UK National Institute for Health and Care Excellence (NICE).^15^ The costs and QALYs after one year were discounted at 3.5% per year.^19^

Costs were initially calculated in £ and converted into €. The incremental QALYs and costs were estimated with a seemingly unrelated regression (SUR) model,^22^ and, like the primary analysis of the clinical outcomes, this was without adjustment for baseline covariates. The estimate of incremental costs and QALYs were then used to report incremental net monetary benefits of the endovascular strategy according to the overall intention to treat population.

Given the moderate rates of non-compliance with trial protocol, and that the non-compliance was not at random, we also applied a complier average causal effects (CACE) model. The CACE estimate reports the potential effect of adhering to trial protocol—endovascular strategy (EVAR if morphologically feasible) or open repair—to provide a less biased estimate of the true causal effect of an endovascular strategy than a per protocol analysis.^23^ ^24^ The CACE approach estimates the causal effects of an endovascular first strategy versus an open repair strategy among those patients who would have complied with the trial protocol for either strategy. Analyses were conducted in the 502 patients with treated ruptures and the CACE estimates for all endpoints, including the incremental net monetary benefits, were reported alongside the intention to treat odds ratio or mean difference in this population at three years (further details in appendix 2).

## Results

### Study population and interventions

The study population has been described previously.^2^Figure 1 shows the 613 randomised patients followed-up to three years after randomisation. Briefly, among the 316 patients in the endovascular strategy group, 275 had aorto-iliac aneurysm rupture, eight had acute symptomatic intact aneurysm, 27 had asymptomatic abdominal aortic aneurysm with other acute diagnoses, and six had other diagnoses. Of the 300/316 who underwent computed tomography, 186 (62%) were considered morphologically suitable for EVAR. Among the 297 patients in the open repair group, 261 had aorto-iliac aneurysm rupture, 14 had acute symptomatic intact aneurysm, 19 had asymptomatic abdominal aortic aneurysm with other acute diagnoses, and three had other diagnoses. In total 536 patients had blood breaching the aneurysm sac (rupture): 34 died before repair and repair was started in 502 (group for principal sensitivity analysis), 259 and 243 in the endovascular strategy and open repair groups, respectively. In 149/259 EVAR was started, and 110/259 patients underwent open repair (26 against protocol, mainly because a staffed endovascular suite was not immediately available). In the open repair group, 33 patients underwent EVAR (mainly because they were poor candidates for general anaesthesia). Between 30 days and three years, two patients in each randomised group emigrated and were lost to follow-up. At randomisation, the mean age was 77, 22% of the patients were women, and the mean aneurysm diameter was 8.4 cm. The baseline characteristics of the 502 treated ruptures, according to randomisation status, were similar to those of the full trial cohort (table 1).

**Figure f1:**
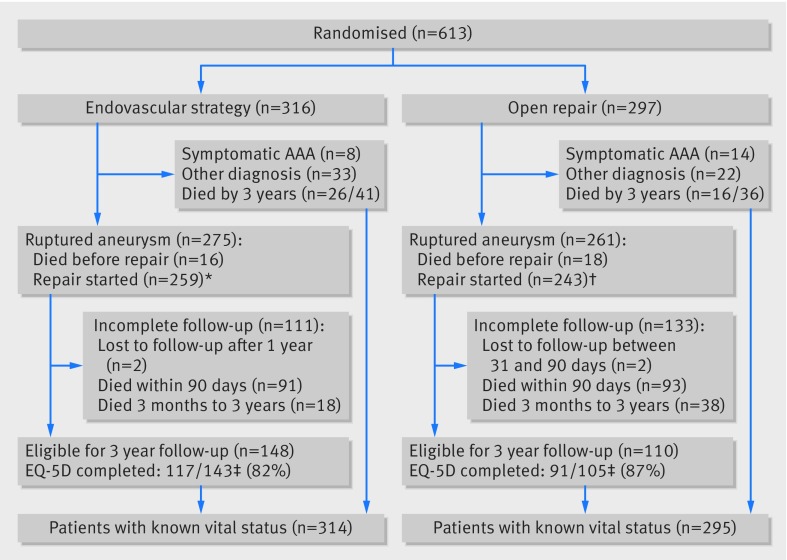
**Fig 1** Flow of patients to three years after randomisation. *Includes 26 patients who had open repairs in breach of protocol; †includes 33 patients who had EVARs in breach of protocol; ‡five patients per randomised group withdrew consent for being contacted about completing EQ-5D questionnaires but allowed their other data to be used. Completion rates reported indicate fully completed questionnaires

**Table 1 tbl1:** Baseline characteristics of patients with ruptured abdominal aortic aneurysm randomised to treatment with endovascular strategy (endovascular repair if aortic morphology is suitable, open repair if not) or open repair. Figures are numbers (percentage) unless stated otherwise^*^

Variable	Endovascular strategy (n=316)	Open repair (n=297)	Rupture repairs	
			Endovascular strategy (n=259)	Open repair (n=243)
Mean (SD) age (years)	76.7 (7.4)	76.7 (7.8)	76.0 (7.4)	76.2 (7.6)
Men	246 (78)	234 (79)	209 (81)	195 (80)
Women	70 (22)	63 (21)	50 (19)	48 (20)
Mean (SD) blood pressure on admission (mm Hg):				
Systolic	110.3 (32.9)	110.5 (31.2)	108.7 (33.1)	109.0 (31.1)
Diastolic	65.3 (21.4)	66.7 (22.5)	65.1 (22.0)	65.3 (22.7)
Hardman index (0-5):				
0	93 (33)	71 (28)	83 (36)	60 (28)
1	130 (46)	124 (48)	103 (44)	97 (46)
2	46 (16)	48 (19)	36 (15)	43 (20)
3	11 (4)	12 (5)	9 (4)	10 (5)
4	2 (1)	2 (1)	2 (1)	2 (1)
5	0 (0)	0 (0)	0 (0)	0 (0)
Computed tomography performed:				
Yes	305 (97)	265 (89)	251 (97)	216 (89)
No	11 (3)	32 (11)	8 (3)	27 (11)
Mean (SD) maximum aortic diameter (cm)^†^	8.5 (1.9)	8.3 (1.8)	8.7 (1.7)	8.4 (1.8)
Mean (SD) neck length (mm)	—	—	24 (17)	23 (16)
Median time (IQR) to repair^‡^ (min)	—	—	47 (28-73)	37 (22-62)

IQR=interquartile range.

*Of 502 in whom rupture repairs was started, data missing for 9 for admission blood pressure, 57 for Hardman index, 68 for maximum aortic diameter, 91 for neck length (91).

†Measured by core laboratory.

‡From randomisation to theatre admission

### Primary outcome: mortality

The mean follow-up for mortality was 4.9 years (median 4.7; range 0.1-7.1 years) with 2.5 mean person years of observation (to death or censoring). There were 179 deaths in the endovascular strategy group and 183 deaths in the open repair group (table 2). The hazard ratio was 0.92 (95% confidence interval 0.75 to 1.13; P=0.41), with similar results for mortality related to aneurysm (0.89, 0.69 to 1.16; P=0.41) and after adjustment (table D in appendix 1). Kaplan-Meier survival curves (fig 2) showed a slight divergence after the acute phase, with lower mortality in the endovascular strategy group between three months and three years (0.57, 0.36 to 0.90; P=0.015), before converging by seven years. The increased number of deaths in the open repair group between three months and three years was not related to the aneurysm (table 2; table D in appendix 1). Subgroup analysis suggested that the endovascular strategy might be more effective in reducing mortality in women than in men (fig A in appendix 3). By three years, 151 (48%) and 165 (56%) patients had died in the endovascular strategy and open repair groups, respectively (odds ratio 0.73, 95% confidence interval 0.53 to 1.00; P=0.053), and the mean life years were 1.72 and 1.61 (P=0.31).

**Table 2 tbl2:** Causes of death in patients randomised to treatment with endovascular strategy (endovascular repair if aortic morphology is suitable, open repair if not) or open repair by group by time period for all randomised patients (n=613)

	Endovascular strategy (n=316)	Open repair (n=297)	Unadjusted hazard ratio (95% CI)	P value
**All follow-up**				
Related to the aneurysm	112	120	0.92 (0.75 to 1.13)	0.41
Cardiovascular	26	23		
Pulmonary	13	15		
Cancer	19	13		
Other	9	12		
Total	179	183		
**0-3 months**				
Related to the aneurysm	104	112	0.98 (0.76 to 1.26)	0.88
Cardiovascular	8	3		
Pulmonary	5	0		
Cancer	1	0		
Other	2	3		
Total	120	118		
**3 months-3 years**				
Related to the aneurysm	5	5	0.57 (0.36 to 0.90)	0.015
Cardiovascular	12	16		
Pulmonary	5	10		
Cancer	7	10		
Other	2	6		
Total	31	47		
**>3 years**				
Related to the aneurysm	3	3	1.44 (0.80 to 2.62)	0.23
Cardiovascular	6	4		
Pulmonary	3	5		
Cancer	11	3		
Other	5	3		
Total	28	18		

**Figure f2:**
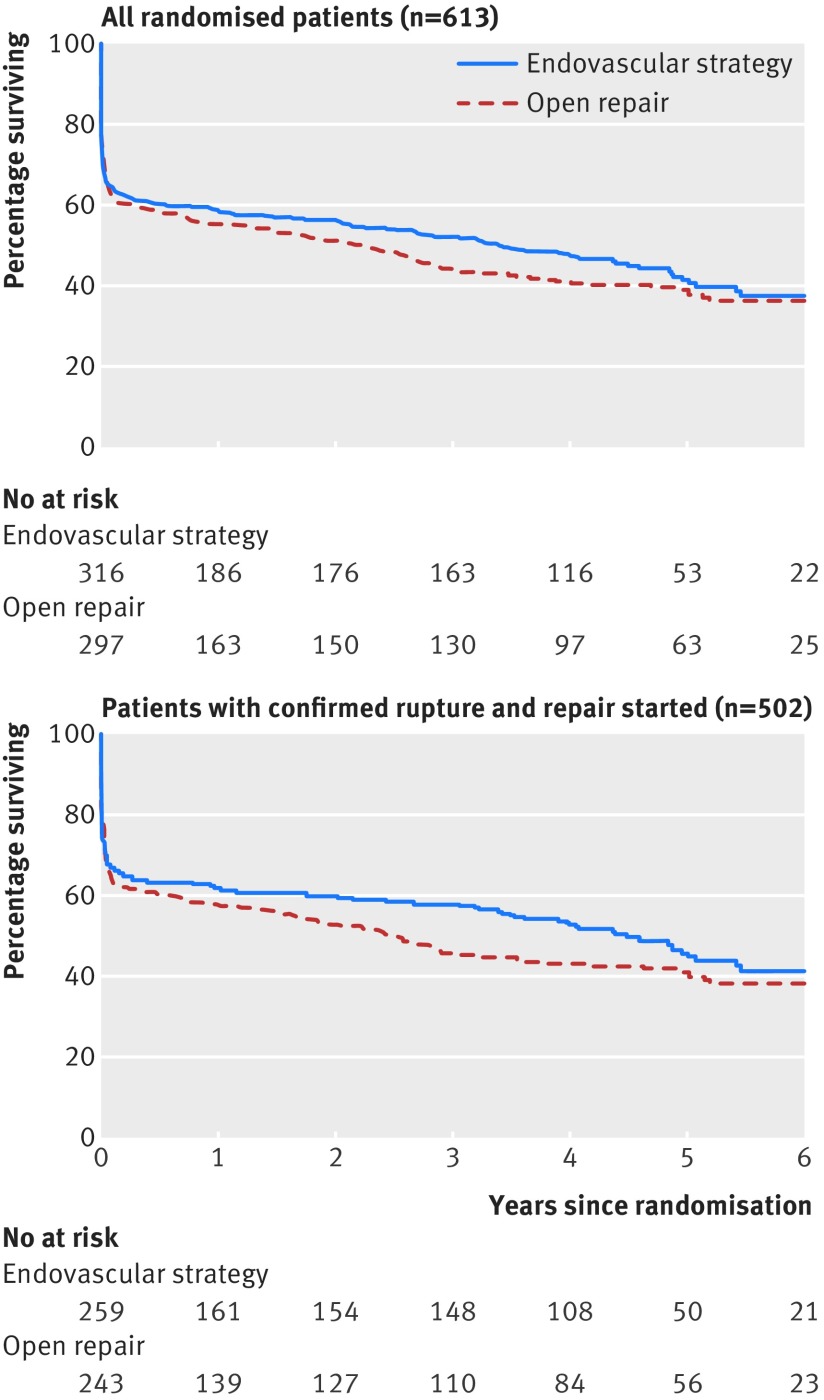
**Fig 2** Kaplan-Meier estimates for overall survival by randomised group (log rank P=0.40 for all 613 randomised patients and P=0.19 for 502 patients with confirmed rupture in whom repair was started)

The Kaplan-Meier curves for the principal sensitivity analysis of 502 treated ruptures followed a similar pattern to the full cohort (fig 2). The overall hazard ratio was 0.86 (95% confidence interval 0.68 to 1.08; P=0.19), which remained similar after adjustment. By three years, 109/259 (42%) and 131/243 (54%) from the endovascular strategy and open repair groups, respectively, had died (odds ratio 0.62, 95% confidence interval 0.43 to 0.88; P=0.008). The odds ratio for a compliers average causal effects (CACE) model was 0.53 (0.34 to 0.84; P=0.008). A post hoc analysis showed that the sex difference was stronger in this subgroup, particularly for deaths related to the aneurysm (hazard ratio 0.44 (95% confidence interval 0.24 to 0.81) in women and 1.09 (0.79 to 1.52) in men; P=0.01 for interaction).

### Secondary outcomes

#### Reinterventions related to aneurysm

Among the 502 treated ruptures, 230 reinterventions related to the aneurysm were recorded within three years of randomisation; 121 and 109 in the endovascular strategy and open repair groups respectively (hazard ratio 1.02, 95% confidence interval 0.79 to 1.32; P=0.88). The reinterventions, categorised by whether they were arterial, related to laparotomy or other, or for a life threatening condition are shown in table E in appendix 1. Overall and by time (acute 0-three months or three months to three years) the reintervention rates were similar between those randomised to an endovascular strategy and those randomised to open repair, with about 28% of each group needing at least one reintervention related to the aneurysm. Figure 3 shows the cumulative incidences for patients with at least one intervention to three years and at least one intervention for a life threatening condition (see also table A in appendix 1). New reinterventions for life threatening conditions continued to occur at a much slower but steady rate between three months and three years in both groups. The hazard ratios for risk of reintervention, both overall and by time, remained similar after adjustment (table F in appendix 1). The indications for mid-term reintervention (both related to the aneurysm and other, by both randomised group and treatment received) between three months and three years are shown in table G in appendix 1: 21% of surviving patients treated with EVAR had a mid-term intervention. Patients and their families ranked amputation as the most adverse reintervention. There were eight amputations within the first three years, five in the endovascular strategy group and three in the open repair group, but seven of these occurred after open repair.

**Figure f3:**
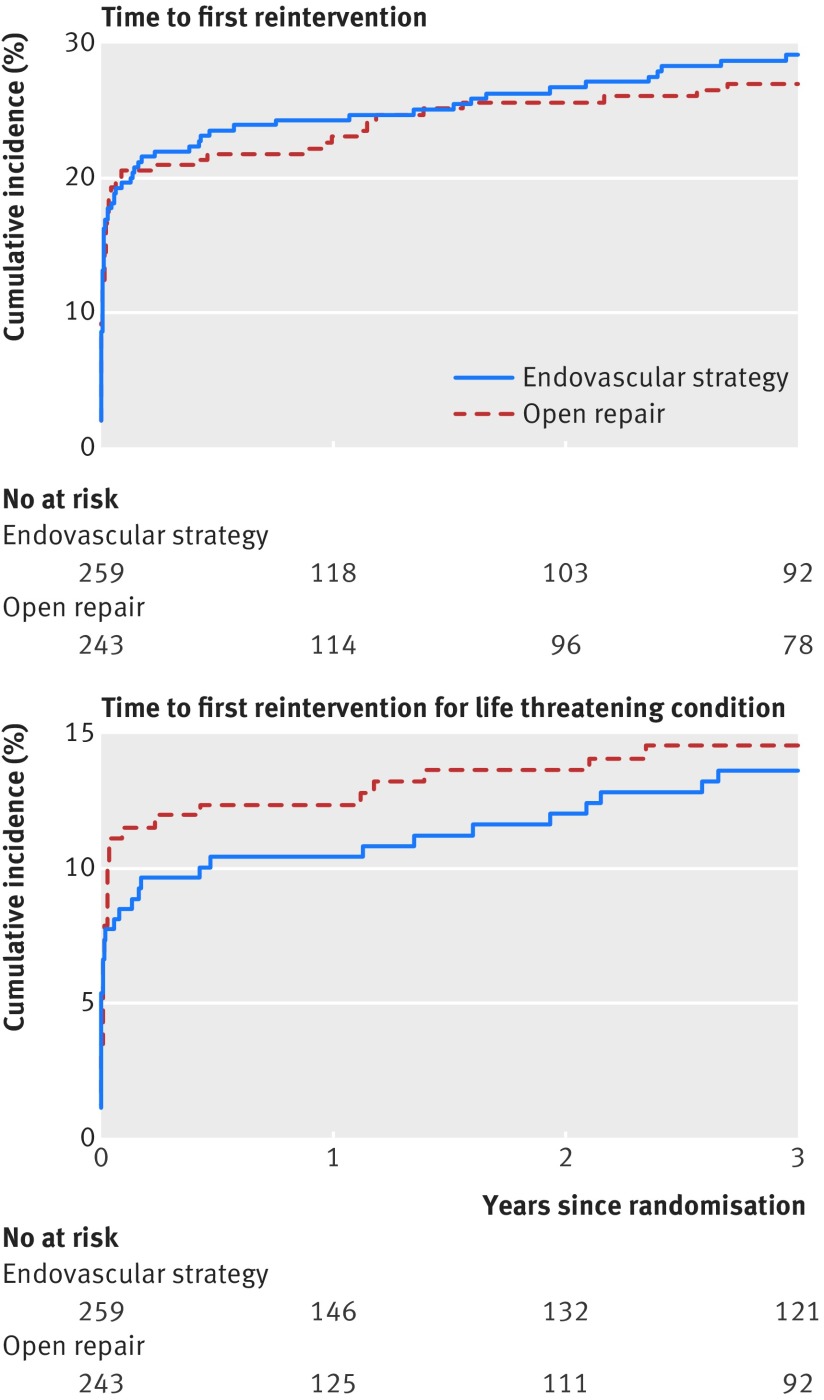
**Fig 3** Cumulative incidence of reinterventions in 502 patients in whom repair of rupture was started. Gray’s test for testing equality of cumulative incidence curves: P=0.643 for time to first reintervention; P=0.713 for time to reintervention for life threatening condition (included hindquarter amputation, colectomy with stoma for mesenteric or colonic ischaemia, graft infection, secondary rupture, and repeat aneurysm repairs (full list in table A in appendix 1)

### Quality of life, QALYs, costs, and cost effectiveness

The average QoL was higher in the endovascular strategy group in the first year but by three years was similar across the randomised groups (fig 4). Table 3 shows QALYs, costs, and cost effectiveness for the full intention to treat population (n=613). The QALY gain at three years for the endovascular strategy group was 0.166 (95% confidence interval 0.002 to 0.331) and was higher for women and those with highest baseline Hardman index but was otherwise similar across subgroups (table H in appendix 1). Resource use up to three years after randomisation, related to primary admission and readmissions related to the aneurysm, including those for reinterventions, are detailed in table I in appendix 1. Overall, patients in the endovascular strategy stayed, on average, fewer days in hospital than those in the open repair group; the mean total days in hospital was 14.4 versus 20.5, with an overall cost reduction of −£2605 (95% confidence interval −£5966 to £702) (−€2816, −€6425 to €794).

**Figure f4:**
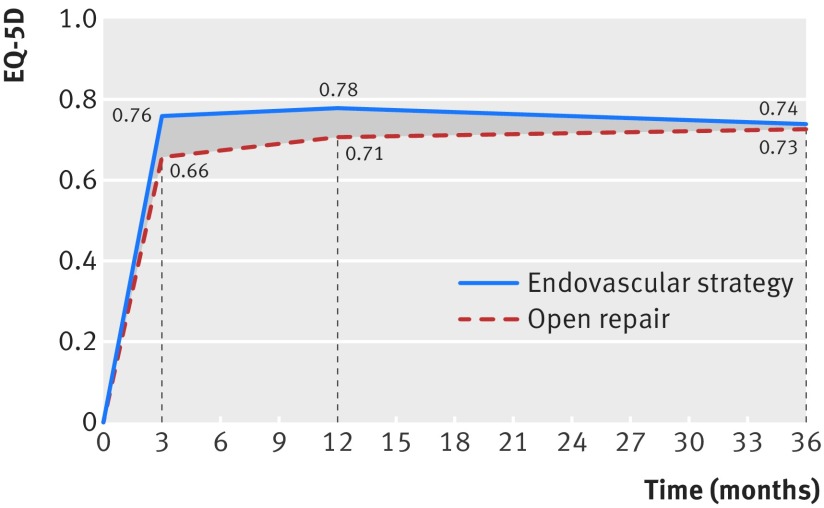
**Fig 4** Mean quality of life (EQ-5D score) by randomised group for 502 patients with repair of rupture started, alive and eligible for follow-up at specified time points. Randomisation of critically ill patients needing urgent surgery to avoid death meant that baseline EQ-5D scores were not obtained and set at zero. Average utility scores shown at 3 months and 1 and 3 years. In endovascular strategy versus open repair group mean difference was 0.097 (95% confidence interval 0.031 to 0.163; P=0.004, n=318) at 3 months; 0.068 (0.002 to 0.134; P=0.045, n=301) at 1 year; and 0.013 (−0.069 to 0.096; P=0.751, n=262) at 3 years

**Table 3 tbl3:** Quality adjusted life years (QALYs), costs, and cost effectiveness at three years for all patients (n=613) randomised to treatment with endovascular strategy (endovascular repair if aortic morphology is suitable, open repair if not). Results are reported after multiple imputation

	Endovascular strategy			Open repair		Mean difference (95% CI)	P value
	No of patients	Mean (SD)		No of patients	Mean (SD)		
Life years	316	1.72 (1.43)		297	1.61 (1.41)	0.115 (−0.110 to 0.341)	0.314
QALYs^*^	316	1.14 (1.03)		297	0.97 (1.02)	0.166 (0.002 to 0.331)	0.048
Total cost (£)	316	16 878 (19 624)		297	19 483 (22 412)	−2605 (−5966 to 702)	0.120
Incremental net benefit (£)^†^	**—**	**—**		**—**	**—**	7637 (1820 to 13 454)	0.005

*Includes patients who died and those without proved rupture, who were assumed to have, on average, same quality of life of elective repair patients.^12^ QALYs between 1 and 3 years discounted with NICE’s recommended discount rate of 3.5%.

†Incremental net benefit for endovascular versus open repair calculated by multiplying mean difference in QALY by NICE’s recommended willingness to pay threshold (£30 000 per QALY gain) and subtracting from this incremental cost.

When the incremental costs and QALYs were represented on the cost effectiveness plane, most (88%) estimates were in the quadrant showing the endovascular strategy as “dominant,” with lower mean costs and higher mean QALYs (fig 5). The incremental net monetary benefit of the endovascular strategy versus open repair (QALY valued at £30 000) was positive at £7367 (95% confidence interval £1829 to £13 454) (€7956, €1930 to €14 530), a finding robust to a range of assumptions, and was similar across subgroups (see table H in appendix 1 and fig B in appendix 3). The probability that the endovascular strategy is more cost effective is above 90% across all willingness to pay thresholds for a QALY gain (fig 6).

**Figure f5:**
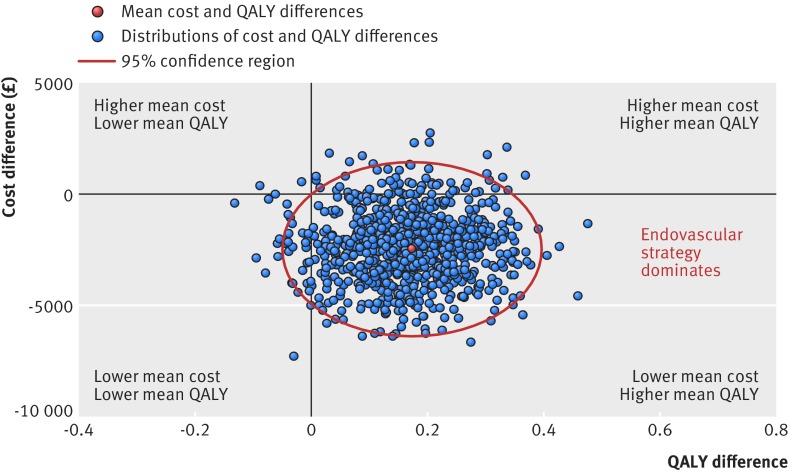
**Fig 5** Uncertainty in mean cost (£) and QALY differences and their joint distribution for endovascular strategy versus open repair for all 613 patients

**Figure f6:**
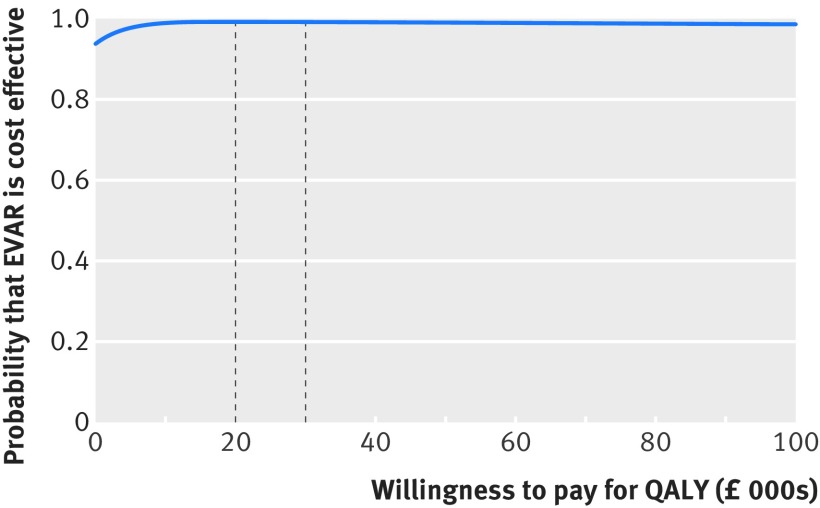
**Fig 6** Cost effectiveness acceptability curve reporting probability that endovascular strategy is cost effective at alternative levels of willingness to pay (£) for QALY gain

We repeated all the above analyses for the 502 patients with confirmed rupture in whom repair was started using both an intention to treat and CACE approach (table 4). Overall, mean differences in EQ-5D, QALYs, and total cost for these patients according to intention to treat were similar to those reported for the full trial cohort (n=613, table 3). In addition to the significant differences in mortality and QALYs, CACE analysis led to larger differences in the cost effectiveness endpoints between treatment groups: the mean incremental net monetary benefit of the endovascular strategy versus open repair was £21 528 (95% confidence interval £5999 to £37 057), almost three times higher than for the full trial cohort and the QALY gains also increased markedly.

**Table 4 tbl4:** Three year outcomes for principal sensitivity analysis of 502 patients with confirmed diagnosis of abdominal aortic aneurysm rupture in whom repair was started. Figures are mean differences unless stated otherwise

Outcome (measure)	No of patients	Estimate (95% CI)		P value^*^
		As randomised (intention to treat)	Complier average causal effect (CACE)	
OR for mortality^†^	498	0.62 (0.43 to 0.88)	0.53 (0.34 to 0.84)	0.008
OR for any reintervention related to the aneurysm	502	1.12 (0.76 to 1.65)	1.16 (0.69 to 1.94)	0.58
EQ-5D^‡^	262	0.013 (−0.069 to 0.096)	0.041 (−0.112 to 0.193)	0.75
QALYs^§^	502	0.229 (0.043 to 0.414)	0.512 (0.084 to 0.940)	0.016
Total cost (£)	502	−2610 (−6200 to 978)	−6126 (−14336 to 2083)	0.154
Incremental net benefit (£)	502	9484 (2828 to 16 140)	21 528 (5999 to 37 057)	0.003

OR=odds ratio.

*Same for intention to treat and CACE estimates, but magnitude of effect might be different.

†4 patients lost to follow-up for mortality by 3 years.

‡For EQ-5D-3L scores, number of patients is total number eligible for follow-up (that is, still alive and not lost to follow-up). EQ-5D missing for 33 (22%) EVAR patients and 21 (19%) open repair patients.

§Includes patients who died.

## Discussion

This is the first randomised comparison of interventions for ruptured abdominal aortic aneurysm with comprehensive mid-term (three year) reporting. We found that after aneurysm rupture an endovascular strategy offers no significant reduction in operative mortality at 30 or 90 days, but there is an interim mid-term survival advantage (three months to three years), which, together with the early gains in quality of life, leads to a mid-term gain in QALYs after three years. Reinterventions related to the aneurysm, particularly those for life threatening conditions, occurred at a similar rate in both groups. The cost differences observed at 30 days (non-significantly in favour of the endovascular strategy group)^2^ were not eroded by an increased burden of reinterventions in later follow-up, and therefore the endovascular strategy is cost effective. All these results are in sharp contrast with those of earlier trials conducted in the elective setting (table 5).

**Table 5 tbl5:** Comparison of mid-term health outcomes from randomised trials of endovascular versus open repair for elective and ruptured abdominal aortic aneurysm repair

Parameter	Elective repair	Rupture repair from IMPROVE trial
30 day mortality	2.5-fold higher for open repair^25^	No difference^2^
3 year mortality	No difference^25^	Endovascular strategy better
Length of primary hospital stay	No difference^26^ ^27^	Shorter for endovascular strategy^12^
Reintervention rate	2-3-fold higher after EVAR^25^ ^27^ ^28^	No difference
Quality of life	Better after open repair or no difference at 1 year^27^ ^29^	Better at 3 months, 1 year for endovascular strategy
Costs	EVAR higher^27^ ^30^	Endovascular strategy less
Cost effectiveness	EVAR not cost effective^27^ ^30^ ^31^	Endovascular strategy cost effective

To deal with criticisms about the pragmatic design of the trial, we also report analyses (both causal and intention to treat) for the 502 patients in whom repair of rupture was started, which emphasise the survival benefit, QALY gain, and cost effectiveness of the endovascular strategy over three years.

### Interpretation

The reasons for these mid-term differences between the comparative effectiveness of an endovascular strategy and open repair in the emergency and elective settings remain speculative. The shock associated with rupture probably kills many patients irrespective of the type of repair, but EVAR is less invasive and can be conducted under local anaesthesia so that patients recover more rapidly than after open repair.

Acute kidney injury is common after repair of a ruptured aneurysm, particularly open repair, and has prolonged consequence for mortality.^32^ ^33^ ^34^ Acute kidney injury was not formally documented, but 46 patients in the open repair group with confirmed rupture required renal replacement therapy postoperatively versus 32 in the endovascular strategy group, suggesting that this might have contributed to the better three year outcomes in the endovascular strategy group. Patients in the open repair group had longer stays in critical care than the endovascular strategy group (average 6.3 versus 4.2 days), and there is some evidence that prolonged stay in critical care is associated with higher long term mortality.^35^

The proportion of women in the IMPROVE trial (22%) was much higher than in the trials of elective aneurysm repair, and the advantages of the endovascular strategy were possibly greater in women than in men. The continuing burden of major reinterventions related to laparotomy after open repair for ruptures (not seen, or not reported, after elective open repair) might contribute to mid-term costs in the open repair group, while endovascular devices and the technical skills to deploy them might have improved since the trials of elective repair. The convergence of the survival curves beyond three years is unexplained too, but this phenomenon also has been observed in the analysis of recent registry data and an earlier analysis from Medicare.^6^ ^36^

### Strengths and limitations

This study has several limitations. Firstly, this was a pragmatic trial in the emergency setting and not all randomised patients (with a clinical diagnosis of rupture) had a ruptured aneurysm, although 99% did have an aneurysm. Secondly, some of the patients with ruptured aneurysm died before repair (similar numbers in each randomised group), and in this emergency setting the non-compliance rate was higher than anticipated (about 10% in each group). Thirdly, though this is by far the largest of the three recent European trials, with hindsight the sample size might have been larger to allow for non-compliance. Fourthly, after 30 days follow-up focused mainly on the group of 502 patients in whom repair of a ruptured aneurysm was started. Such patients, however, are the clinically most relevant group and were analysed both by intention to treat and complier average causal estimates for all outcomes. Fifthly, after the acute period, data on reinterventions were limited only to those related to the aneurysm and its repair; however, these data were complete, including procedures related to the aneurysm at hospitals outside the trial. Finally, as there was no blinding it also is possible that patients who received EVAR might have reported better quality at early time points.

There also are several strengths to this study. Firstly, recruitment was non-selective, and over half the potentially eligible patients at the trial centres were randomised,^2^ increasing the generalisability of the findings. Secondly, it is the first prospective randomised study with complete clinical and health economic mid-term follow-up. Thirdly, few patients were lost to follow-up, and data completion rates were excellent.

### Summary

This mid-term follow-up provides convincing support for the benefits of an endovascular strategy (EVAR if morphologically feasible) versus open repair to treat patients with ruptured abdominal aortic aneurysm. At three years, the endovascular strategy offers an increase in QALYs, without an excess of reinterventions, and is cost effective.

**What is already known on this topic**

The overall mortality associated with ruptured abdominal aortic aneurysm remains high
Individual patient data meta-analysis of three recent European randomised trials has shown that the use of keyhole endovascular repair (compared with traditional open repair) does not reduce the high acute mortality (0-90 days) from emergency surgery

**What this study adds**

This is the first randomised trial comparing the use of the keyhole endovascular aneurysm repair versus traditional open surgery with comprehensive mid-term outcomes, including reinterventions, quality of life, costs, and evaluation of cost effectiveness
A combination of mid-term survival advantage with early gains in quality of life led, after three years, to significantly higher QALYs in the endovascular strategy group, which was achieved without an excess of reinterventions and further hospital costs
An endovascular strategy (endovascular repair when morphologically feasible) is both clinically effective and cost effective and should be adopted more widely

## Web Extra

Extra material supplied by the author

Appendix 1: Supplementary tables A-J

Appendix 2: Additional methods and statistical information

## Notes

We thank all the patients and their families and/or carers who have participated in or contributed to this trial. IMPROVE Writing Committee P Ulug, J T Powell, R M Greenhalgh (Vascular Surgery Research Group, Imperial College, London); M J Sweeting, S G Thompson (Cardiovascular Epidemiology Unit, Department of Public Health and Primary Care, University of Cambridge); M Gomes, R D Grieve (Department of Health Services Research and Policy, London School of Hygiene and Tropical Medicine); R J Hinchliffe (Bristol Centre for Surgical Research, University of Bristol); M M Thompson (St George’s Hospital, London); R Ashleigh (Department of Radiology, University Hospital of South Manchester). IMPROVE Trial Investigators Grant applicants: Janet T Powell (chief investigator), Bruce Braithwaite, Nicholas J Cheshire, Roger M Greenhalgh, Richard D Grieve, Tajek B Hassan, Robert J Hinchliffe, Simon Howell, Fionna Moore, Anthony A Nicholson, Matt M Thompson, Simon G Thompson. Data and trial management: Pinar Ulug (trial manager). Statistical analyses: Michael J Sweeting and Simon G Thompson. Health economics costs analyses: Manuel Gomes and Richard J Grieve. Trial management committee: Janet T Powell (chair), Ray Ashleigh, Manuel Gomes, Roger M Greenhalgh, Richard Grieve, Robert J Hinchliffe, Michael Sweeting, Matt M Thompson, Simon G Thompson, Pinar Ulug. Trial steering committee: Ian Roberts (chair), Peter R F Bell, Anne Cheetham, Alison Halliday. Data monitoring and ethics committee: Charles Warlow (chair), Peter Lamont, Jonathan Moss, Jan Tijssen. Local principal investigators (in order of site start date from earliest to most recent); numbers in parentheses indicate the number of patients entered into the trial: UK: Colin D Bicknell, Nicholas J Cheshire (to Oct 2014), Imperial College Healthcare NHS Trust, London (20); Jonathan R Boyle, Addenbrooke’s Hospital, Cambridge (40); Ferdinand Serracino-Inglott, J Vince Smyth (Dec 2012-Nov 2013), Manchester Royal Infirmary, Manchester (69); Matt M Thompson, Robert J Hinchliffe, St George’s Hospital, London (75); Rachel Bell, Guy’s and St Thomas’ Hospital, London (81); Noel Wilson, Kent and Canterbury Hospital, Canterbury (23); Matt Bown, Martin Dennis (to Dec 2010), Leicester Royal Infirmary, Leicester(18); Meryl Davis, Royal Free Hospital, London (1); Ray Ashleigh, University Hospital of South Manchester, Manchester (21); Simon Howell, Leeds General Infirmary, Leeds (23); Michael G Wyatt, Freeman Hospital, Newcastle (23); Domenico Valenti, King’s College Hospital, London (2); Paul Bachoo, Aberdeen Royal Infirmary, Aberdeen (4); Paul Walker, James Cook University Hospital, Middlesbrough (5); Shane MacSweeney, Queen’s Medical Centre, Nottingham (34); Jonathan N Davies, Royal Cornwall Hospital, Truro (5); Dynesh Rittoo, Simon D Parvin (to Dec 2011), Royal Bournemouth Hospital, Bournemouth (22); Syed Waquar Yusuf, Royal Sussex County Hospital, Brighton (5); Colin Nice, Queen Elizabeth Hospital, Gateshead (5); Ian Chetter, Hull Royal Infirmary, Hull (32); Adam Howard, Colchester General Hospital, Colchester (24); Patrick Chong, Frimley Park Hospital, Surrey (14); Raj Bhat, Ninewells Hospital, Dundee (8); David McLain, Royal Gwent Hospital, Newport; Andrew Gordon, Ian Lane (to Jun 2012), University Hospital of Wales, Cardiff (4); Simon Hobbs, New Cross Hospital, Wolverhampton (3); Woolagasen Pillay, Doncaster Royal Infirmary, Doncaster (8); Timothy Rowlands, Amin El-Tahir (to Nov 2012), Royal Derby Hospital, Derby (13); John Asquith, Royal Stoke University Hospital, Stoke-on-Trent (15); Stephen P Cavanagh, The York Hospital, York (3). Canada: Luc Dubois, Thomas L Forbes (to Aug. 2014), London Health Sciences Centre and Western University, London, Ontario (13). Trial coordinators: Emily Ashworth, Sara Baker, Hashem Barakat, Joanne Brown, Debbie Campbell, Tina Chance, Angela Chrisopoulou, Andrea Croucher, Leela Dabee, Nikki Dewhirst, Christopher Fenner, Andy Gibson, Siobhan Gorst, Norma Gourlay, Lynne Graves, Josie Hatfield, Cían Hughes, Elizabeth Keene, Michelle Lapworth, Teresa Novick, Daré Oladokun, Gareth Owen, Noala Parr, Joanna Smee, Helen Thompson, Orla Thunder, Sue Ward, Lesley Wilson, Janet Woods.

Ethical approval: The study was approved by South-Central Berkshire research ethics committee 08/H0505/173 (England and Wales), Scotland A research ethics committee 08/MRE00/90 (Scotland), and the University of Western Ontario health sciences research ethics board 17698 (Canada). There was a two stage consent procedure: brief initial consent and full postoperative consent. The National Information Governance Board (ECC 4-03 (f) 2012) approved the collection of mortality details for patients who died before second postoperative consent. Results were reviewed at regular intervals by an independent data monitoring and ethical committee.

Data sharing: Patient level data can be made available from the corresponding author or statistician (MJS) after discussion with the trial management committee. Consent from participants for data sharing was not obtained but any shared data will be anonymised.

Transparency: The lead author affirms that this manuscript is an honest, accurate and transparent account of the study being reported; that no important aspects of the study have been omitted; and that any discrepancies from the study as planned have been explained.
