# Supplementary material for: Comparative clinical effectiveness and cost effectiveness of endovascular strategy v open repair for ruptured abdominal aortic aneurysm: three year results of the IMPROVE randomised trial
Source: BMJ. 2017 Nov 14;359:j4859. doi: 10.1136/bmj.j4859 (PMC5682594; doi:10.1136/bmj.j4859)
Supplement: Supplementary file 1 — Appendix 1: Supplementary tables A-J [file pulu039133.ww1.pdf]

Appendix 1: Supplementary Tables [posted as supplied by author]

Table A Aneurysm-related re-interventions for life-threatening conditions. These are based on the scoring system developed for the EVAR 1 trial (8), with additional re-interventions scored by IMPROVE trial investigators.

**Procedures for life-threatening aneurysm-related complications**
Conversion of failed EVAR to open repair
Axillo-bifemoral bypass for graft infection or aortic occlusion
Re-operation of open or endovascular repair for graft infection, aneurysm extension above or below the original graft, secondary rupture or other reasons
Endovascular repair of proximal (type 1a endoleak) or distal (type 1b endoleak)
First laparotomy for abdominal compartment syndrome
Hindquarter amputation
Colectomy ± stoma formation for mesenteric or colonic ischaemia in 1° or later admission

Other aneurysm-related procedures included endovascular procedures to the aorta including stapling, ligation, sclerosis or embolization of type 2 endoleaks, femoro-femoral cross-over grafting, distal graft limb revascularisation (open or endovascular), lower limb revascularisation (open or endovascular), leg amputation (above, through or below knee), incisional hernia repair, laparotomy for adhesions or small bowel obstruction, access site repairs and other minor procedures.

Table B Source of]re-intervention data in 502 patients with repair of rupture started.

**Time period re-intervention**	**Principal source according to protocol**	**Primary source of re-intervention data**				**No. re-interventions identified**
		**Trial co-ordinator**	**HES***	**Patient questionnaires**	**Audit**	
**0-1 year**	**Trial co-ordinator**	**162**	**5**	2	**22**	**191,**<br>with 0 at non-trial hospitals
**1-3 years**	**Audit, supported by HES**	**20**	**10**	1	**17**	**48**<br>with 2 at non-trial hospitals

*Health Episode Statistics for England only, providing data for trial and non-trial hospitals to 3 years after patient discharge from primary admission. During the first year, all data from HES or questionnaires were subsequently verified by the trial co-ordinators: there were an additional 6 patients with other surgery not related to the AAA. Between 1 and 3 years all data from HES or questionnaires were verified by audit. Data from the trial co-ordinators were verified by audit. Audit also identified patients with first and subsequent re-interventions beyond 3 years.

Table C Variables considered for multiple imputations

Variable	Missing values[a] N (%)	Imputation model
Baseline variables and vital status[a]		
Loss of consciousness during care episode	27 (4%)	Logistic regression
Admission haemoglobin	6 (1%)	Predictive mean matching
Admission creatinine	13 (2%)	Predictive mean matching
Acute myocardial ischaemia	52 (8%)	Logistic regression
Lowest recorded systolic blood pressure	46 (8%)	Predictive mean matching
Lowest recorded diastolic blood pressure	53 (9%)	Predictive mean matching
Maximum aortic diameter[b]	95 (15%)	Predictive mean matching
Aneurysm neck diameter[b]	230 (38%)	Predictive mean matching
Aneurysm proximal neck angle[b]	132 (22%)	Predictive mean matching
Aneurysm neck length[b]	132 (22%)	Predictive mean matching
Death within 3 years	4 (1%)	None done
Resource use variables[a]		
Primary admission - Time in theatre	22 (4%)	Predictive mean matching
Primary admission - Days in critical care	13 (2%)	Predictive mean matching
Primary admission - Days in routine ward	48 (8%)	Predictive mean matching
Re-admissions up to 36months - days in critical care	32 (11%)	Predictive mean matching
Re-admission up to 36 months- days in routine ward	31 (10%)	Predictive mean matching
Outpatient visits at 12 months	47 (16%)	Predictive mean matching
Family doctor visits at 12 months	83 (28%)	Predictive mean matching
Nurse at home visits at 12 months	68 (23%)	Predictive mean matching
Quality-of-life (QoL) variables		
EQ-5D at 3 months	66 (21%)	Predictive mean matching
EQ-5D at 12 months	72 (24%)	Predictive mean matching
EQ-5D at 36 months	50 (19%)	Predictive mean matching

[a]For baseline variables, vital status and primary admissions, the overall sample size was all randomised patients (n=613). Baseline EQ-5D was neither obtained nor imputed in critically ill patients requiring urgent surgery to avoid death. For other resource use and QoL variables, the relevant sample sizes were those patients eligible for the 3, 12 and 36 month follow-up (n=318, 3 months, n=301, 12 months, and n=258, 36 months respectively). [b]conditional on ruptured AAA, symptomatic AAA or Incidental AAA in final diagnosis..

Table D Unadjusted and adjusted hazard ratios for endovascular strategy versus open repair, for both all-cause and aneurysm-related mortality in the full trial cohort (n=613)

Overall

	N	Hazard Ratio (95% CI)	p-value
**All-cause mortality**			
Unadjusted	613	0.92 (0.75, 1.13)	0.41
*Adjusted (complete-cases)	506	0.91 (0.72, 1.14)	0.41
*Adjusted (multiply imputed)	613	0.90 (0.73, 1.10)	0.31
**Aneurysm-related mortality**			
Unadjusted	613	0.90 (0.69, 1.16)	0.41
*Adjusted (complete-cases)	506	0.89 (0.67, 1.19)	0.44
*Adjusted (multiply imputed)	613	0.88 (0.68, 1.15)	0.35

*Adjusted for age, sex, Hardman index, and lowest systolic blood pressure.

By time period

	No. remaining under follow-up	Unadjusted Hazard Ratio (95% CI)	p-value
**All-cause mortality**			
0-3 months	613	0.98 (0.76, 1.26)	0.88
>3 months – 3 years	373	0.57 (0.36, 0.90)	0.015
>3 years	293	1.44 (0.80, 2.62)	0.23
**Aneurysm-related mortality**			
0-3 months	613	0.90 (0.69, 1.17)	0.43
>3 months – 3 years	373	0.88 (0.26, 3.06)	0.85
>3 years	293	0.96 (0.19, 4.78)	0.96

Table E Re-interventions by randomised group according to time period and if for a life-threatening condition in 502 patients with repair of rupture started.

	Ruptured AAA patients who commenced operation N=502	
**AAA-related re-intervention**	**EVAR strategy**	**Open repair**
No. patients with ≥1 re-intervention	75 / 259 (29%)	66 / 243 (27%)
No. re-interventions/p-years (rate per 100 person-years)	121/463.8 (26.0)	110/387.3 (28.4)
Arterial<br>Laparotomy<br>Other	93 (77%)<br>16 (13%)<br>12 (10%)	63 (57%)<br>34 (31%)<br>13 (12%)
For a life-threatening arterial-related condition<br>Yes<br>no	 <br>37 (40%)<br>56 (60%)	 <br>40 (63%)<br>23 (37%)
For a life-threatening laparotomy-related condition<br>Yes<br>No	 <br>5 (31%)<br>11 (69%)	 <br>4 (12%)<br>30 (88%)
**Randomisation to 3 months**		
No. patients with at least one re-intervention	57/259 (22%)	52/243 (21%)
No. re-interventions/p-years (rate per 100 person-years)	81/43.6 (185.6)	89/38.9 (228.6)
Arterial<br>Laparotomy<br>Other	60 (74%)<br>11 (14%)<br>10 (12%)	50 (56%)<br>27 (31%)<br>12 (14%)
For a life-threatening arterial-related condition<br>Yes<br>no	 <br>26 (43%)<br>34 (57%)	 <br>33 (66%)<br>16 (34%)
For a life-threatening laparotomy-related condition<br>Yes<br>No	 <br>2 (18%)<br>9 (82%)	 <br>3 (11%)<br>24 (89%)
**> 3 months – 3 years**		
No. patients with at least one re-intervention	27/167 (16%)	15/146 (10%)
No. re-interventions/p-years (rate per 100 person-years)	40/420.1 (9.5)	21/348.3 (6.0)
Arterial<br>Laparotomy<br>Other	33 (83%)<br>5 (13%)<br>2 (5%)	13 (62%)<br>7 (33%)<br>1 (5%)
For a life-threatening arterial-related condition<br>Yes<br>no	 <br>11 (33%)<br>22 (67%)	 <br>7 (54%)<br>6 (46%)
For a life-threatening laparotomy-related condition<br>Yes<br>No	 <br>3 (60%)<br>2 (40%)	 <br>1 (14%)<br>6 (86%)

Table F Unadjusted and adjusted hazard ratios for EVAR strategy versus open repair, for time to first AAA re-intervention and time to any reintervention within the first 3-years of follow-up for 502 patients with repair of rupture started.

Rate per 100 person-years (no./p-years)		Model	N	Hazard Ratio (95% CI)	p-value
**Time to first aneurysm-related re-intervention**					
**EVAR strategy**	**Open repair**				
22.6 (75/331.5)	20.8 (65/313.1)	Unadjusted	502	1.11 (0.80, 1.55)	0.53
		*Adjusted (complete-cases)	343	1.08 (0.73, 1.62)	0.70
		*Adjusted (multiply imputed)	502	1.12 (0.80, 1.56)	0.51
**Time to any aneurysm-related re-intervention (Anderson-Gill model)**					
**Overall**					
**EVAR strategy**	**Open repair**				
26.1 (121/463.8)	28.1 (109/387.3)	Unadjusted	502	1.02 (0.79, 1.32)	0.88
		*Adjusted (complete-cases)	343	0.92 (0.68, 1.26)	0.62
		*Adjusted (multiply imputed)	502	1.04 (0.80, 1.35)	0.79
**0-3 months**					
**EVAR strategy**	**Open repair**				
185.6 (81/43.6)	226.1 (88/38.9)	Unadjusted	502	0.88 (0.65, 1.19)	0.40
		*Adjusted (complete-cases)	343	0.78 (0.55, 1.11)	0.16
		*Adjusted (multiply imputed)	502	0.88 (0.65, 1.20)	0.43
**>3 months – 3 years**					
**EVAR strategy**	**Open repair**				
9.5 (40/420.1)	6.0 (21/348.3)	Unadjusted	313	1.57 (0.93, 2.67)	0.09
		*Adjusted (complete-cases)	217	1.67 (0.82, 3.43)	0.16
		*Adjusted (multiply imputed)	313	1.54 (0.90, 2.62)	0.12

*Adjusted for age, sex, Hardman index, lowest systolic blood pressure, neck length.

Table G Indications for midterm re-interventions (between 3 months and 3 years) in 502 patients with repair of rupture started.

**Aneurysm-related indications For 313 repairs with rupture started who survived beyond 90 days**	Randomised to EVAR strategy N=167	Randomised to open repair N=146	EVAR N=125	Open repair N=188
Access site	3	1	2	2
Bowel ischaemia	2	1	0	3
Distal aneurysm	3	4	3	4
Endograft kinking	2	2	4	0
Endograft migration	1	0	1	0
Endoleak*	14	2	16	0
False aneurysm	1	0	1	0
Graft thrombosis/occlusion	6	1	4	3
Graft infection - aorta	0	2	0	2
Graft infection – fem-fem	0	0	0	0
Incisional hernia	0	3	0	3
Limb ischaemia	2	2	3	1
Ostomy	0	1	0	1
Proximal aneurysm	1	0	1	0
Secondary rupture**	2	0	2	0
Symptomatic adhesions	1	1	0	2
**Other indications**				
Nutritional support	0	1	0	1
Renal failure	1	0	1	0
**Total re-interventions**	39 in 27 patients	21 in 15 patients	38 in 26 patients	22 in 16 patients

* 4 type 1A (one also had type 2), 2 type 1B (one also had type 2), 9 type 2 only and 1 type 3; ** one patient with a type IA endoleak and renal failure requiring dialysis and one with a type 1B endoleak.

In 3 patients two indications were treated in the same session, one for bowel ischaemia and incisional hernia, one for type 1A endoleak and fistula formation for renal dialysis and one for type 1B endoleak and common iliac aneurysm.

In those randomised to the EVAR strategy, 17/39 of the re-interventions were for a life-threatening condition versus 7/21 in those randomised to open repair. In those having undergone EVAR 14/38 of the re-interventions were for life-threatening conditions versus 10/22 in those having undergone open repair.

Table H Incremental costs (£ GBP), QALYs and net monetary benefits [95% CI] (£ GBP) within 3-years of randomisation, by subgroups, at NICE* recommended willingness to pay threshold for a QALY gain (£30,000 per QALY) in the total trial cohort (n=613).

*NICE National Institute for Health and Care Excellence

Subgroup		Incremental cost [95% CI]	Incremental QALY [95% CI]	Incremental net monetary benefit (INB) [95% CI][a]	Interaction p-value for INB
Sex	Male (N=480)	-4066 [-7573, -557]	0.092 [-0.099, 0.283]	6824 [204, 13444]	p= 0.440
	Female (N=133)	2817 [-3806, 9439]	0.358 [0.038, 0.712]	7914 [-4401, 20229]	
Hardman Index	Hardman=0 (N=164)	-4017 [-9758, 1725]	0.016 [-0.141, 0.465]	8878 [-1686, 19442]	p=0.279
	Hardman=1 (N=254)	-2448 [-7022, 2126]	0.005 [-0.244, 0.254]	2594 [-5943, 11131]	
	Hardman≥2 (N=121)	-923 [-7306, 5461]	0.423 [0.092, 0.755]	13622 [2025, 25219]	
Neck length	Length < 22 (N=234)	-769 [-5595, 4057]	0.216 [-0.027, 0.459]	7243 [-1216, 15702]	p=0.461
	Length ≥ 22 (N=247)	-4250 [-8859, 358]	0.111 [-0.131, 0.352]	7568 [-959, 16095]	
Lowest SBP	SBP < 90 (N=263)	-3881 [-8604, 842]	0.067 [-0.185, 0.319]	5881 [-2949, 14711]	p=0.275
	SBP ≥ 90 (N=305)	-1540 [-5879, 2798]	0.213 [-0.013, 0.439]	7932 [-14.2, 15878]	

Table I Resource use, costs (£ GBP) and incremental net monetary benefits (£ GBP) up to 3 years, reported across all patients randomised (n=613). Mean (SD) unless stated. Results are reported after multiple imputation.

	**Resource use**		**Cost (£)**	
	Endovascular strategy (n=316)	Open repair (n=297)	Endovascular strategy (n=316)	Open repair (n=297)
**Between randomisation and 1 year**				
**Primary admission**				
Days in critical care	5.3 (12.0)	7.4 (11.9)	6672 (16430)	9674 (16446)
Days on routine ward	7.0 (11.9)	7.8 (12.0)	1835 (3107)	2031 (3158)
Other resource use[a]			6563 (3779)	4757 (2896)
Transfer to another hospital, N (%)	10 (3%)	36 (12%)		
Number of inpatient days	0.7 (4.5)	4.8 (21.1)	175 (1159)	1245 (5479)
Total days in primary admission	13.0 (20.5)	20.0 (31.9)	15245 (18356)	17707 (20842)
**Re-admissions**				
Re-admissions ≥ 1, N (%)	27 (9%)	11 (4%)		
Number of inpatient days	0.7 (4.6)	0.2 (1.5)	215 (1469)	57 (433)
**Re-interventions**[b]				
Re-interventions ≥ 1, N (%)	64 (20%)	60 (20%)	516 (1315)	655 (1711)
**Outpatient and community care**[c]			525 (739)	829 (5772)
**Total days in hospital up to 1 year**	13.7 (21.3)	20.2 (31.9)	16501 (19084)	19248 (22365)
**Between 1 and 3 years**				
**Re-admissions**				
Re-admissions ≥ 1, N (%)	24 (8%)	12 (4%)		
Number of inpatient days	0.7 (4.4)	0.3 (2.3)	270 (1667)	137 (981)
**Re-interventions**				
Re-interventions ≥ 1, N (%)	20 (6%)	11 (4%)	107 (626)	98 (690)
**Total days in hospital up to 3 years**	14.4 (23.4)	20.5 (32.1)	16878 (19624)	19483 (22412)
**Incremental cost** (£) [95% CI]			-2605 [-5966, 702]	
**Incremental net monetary benefit** (£)[d]			7637 [1820, 13454]	

[a]Includes costs related to emergency room, CT scan, devices, consumables and theatre (see table J for sources). [b] Includes six re-interventions that were not related to AAA which is why the numbers reported here (n=124) differ to those for tables B and E (n=118). Also it should be noted that an individual patient can have a reintervention in both the time period between randomisation and 1 year and between 1 and 3 years. Hence simply summing the number of reinterventions across the two time periods would not give the same total number of re-interventions as in Table B. [c]Includes costs related to outpatient, GP and district nurse visits. [d]The INB for endovascular vs. open repair is calculated by multiplying the mean difference in QALY by NICE's recommended willingness-to-pay threshold (£30,000 per QALY gain) and subtracting from this the incremental cost.

Table J Unit costs (£ GBP) for analyses in table I

Description	Unit	Open repair	Endovascular strategy	Source
Medical devices & parts				
Endovascular stent and parts	Patient		5 700	Medtronic©, Cook Medical©[a]
Vascular graft (straight)	Patient	623		Maquet©
Vascular graft (bifurcated)	Patient	901		Maquet©
Consumables				
Endovascular package	Patient		600	Maquet©, Cook Medical©
Mechanical retractor	Patient	90		Health-Care Equipment©
Cell Salvage	Patient	74		Davies et al 2006[9]
Surgical instrument set	Patient	51	51	HealthCare Equipment©
Anaesthetics & other drugs	Patient	184	41	British National Formulary 2012[b]
Contrast agent	ml	0.10	0.10	IMPROVE centres[b]
Blood	unit	132	132	NHS Blood & Transplant 2012
Platelets	unit	205	205	NHS Blood & Transplant 2012
Fresh frozen plasma	unit	25	25	NHS Blood & Transplant 2012
CT scan	unit	105	105	NHS reference costs 2012
Emergency room	Minute	0.40	0.40	Dixon et al 2009[10]
Overheads				
Theatre	Minute	2.65	2.65	IMPROVE centres
Staff[c]				
Surgeon (consultant)	Minute	2.20	2.20	PSSRU 2012 [Curtis 2012] [11]
Surgeon (registrar)	Minute	1.16	1.16	PSSRU 2012
Anaesthetist (consultant)	Minute	2.20	2.20	PSSRU 2012
Anaesthetist (registrar)	Minute	1.16	1.16	PSSRU 2012
ODA	Minute	0.58	0.58	PSSRU 2012
Scrub Nurse	Minute	0.72	0.72	PSSRU 2012
Runner	Minute	0.58	0.58	PSSRU 2012
Senior House Officer	Minute	0.83		PSSRU 2012
Radiologist (consultant)	Minute		2.20	PSSRU 2012
Radiologist (registrar)	Minute		1.16	PSSRU 2012
Radiographer	Minute		0.58	PSSRU 2012
Radiologist Nurse	Minute		0.72	PSSRU 2012
Critical care				
ITU/HDU – 1 organ supported	Bed-day	630	630	NHS reference costs 2012 (DH 2012)
ITU/HDU – 2 organs supported	Bed-day	870	870	NHS reference costs 2012<br>refeence costs 2010-2011
ITU/HDU – 3 organs supported	Bed-day	1214	1214	NHS reference costs 2012
ITU/HDU – 4 organs supported	Bed-day	1410	1410	NHS reference costs 2012
ITU/HDU – 5 organs supported	Bed-day	1587	1587	NHS reference costs 2012
ITU/HDU – 6 organs supported	Bed-day	1759	1759	NHS reference costs 2012
ITU/HDU – 7 organs supported	Bed-day	2000	2000	NHS reference costs 2012
Other hospital care				
Inpatient Coronary care unit	Bed-day	436	436	NHS reference costs 2012
Inpatient Stroke unit	Bed-day	309	309	NHS reference costs 2012
Inpatient Routine ward[d]	Bed-day	260	260	NHS reference costs 2012
Outpatient doctor visit	visit	139	139	PSSRU 2012
Outpatient nurse visit	visit	85	85	PSSRU 2012
Outpatient Haemodialysis	session	65	65	NHS Blood & Transplant 2012
Community care				
Nursing home	Bed-day	105	105	PSSRU 2012
Family doctor visit[e]	visit	55	55	PSSRU 2012
Nurse at home visit[e]	visit	18	18	PSSRU 2012

[a]Average (range from £5 400 to £6 500) list price of the EVAR stents and parts most supplied to NHS Hospitals for ruptured AAA (Medtronic Endurant and Cook Medical Zenith Flex). [b]Local and general anaesthesia components were taken from one IMPROVE centre. [c]Typical levels of staff use in theatre were recorded in 10 IMPROVE centres. [d]Same tariff was applied to routine ward in both primary and secondary hospitals. [e]Assuming 15-minute appointments.
