# Supplementary material for: Comparative clinical effectiveness and cost effectiveness of endovascular strategy v open repair for ruptured abdominal aortic aneurysm: three year results of the IMPROVE randomised trial
Source: BMJ. 2017 Nov 14;359:j4859. doi: 10.1136/bmj.j4859 (PMC5682594; doi:10.1136/bmj.j4859)
Supplement: Supplementary file 2 — Appendix 2: Additional methods and statistical information [file pulu039133.ww2.pdf]

Appendix 2: Additional methods & statistical information [posted as supplied by author]

Sample size

The primary outcome was mortality, first assessed at 30-days after randomisation. The trial, comparing the groups as randomised, had more than 90% power to detect (as significant at 5%) a difference in 30-day mortality of 14% with 600 patients enrolled. This was based on estimated 30-day mortalities of 47% and 21% for patients receiving open repair and EVAR respectively, an estimate of 55% of patients being anatomically suitable for EVAR after computerised tomography and that 5% of both randomized groups would not have a proven diagnosis of ruptured abdominal aortic aneurysm. Hence estimated 30-day mortality was 44.7% in the open repair group and 30.4% in the endovascular strategy group.

Multiple imputation models

Missing data in baseline covariates, resource use and QoL variables were handled with multiple imputation (MI) using chained equations (1). Under this approach each variable was imputed conditional on fully observed baseline variables such as age and gender, and all other imputed variables. All analyses were stratified by randomised group. The MI procedure was conducted in an integrated way, i.e. model specification and variables included in the imputation models were the same for imputing baseline covariates, resource use and QoL variables. Table S3 reports all the variables considered for multiple imputation, and for each variable, the number of missing values, and the imputation model chosen.

For patients with ruptured AAA patients for whom aneurysm repair was commenced, missing resource use components were imputed from those ruptured AAA patients with observed resource use data. Patients who did not have a ruptured AAA and had no information recorded on being in critical care, were assumed to stay on a routine ward for their entire hospital stay.

Patients with proven ruptured AAA who failed to return the QoL questionnaire administered at 3, 12 or 36 months, had their EQ-5D scores imputed from other ruptured AAA survivors. Hence, these imputations did not use information from those patients who had died prior to either time point (who were assigned an EQ-5D score of zero), those ruptured AAA patients who did not have a repair commenced, or from patients who had symptomatic AAA. For patients who were alive but otherwise ineligible for follow-up, since 99% of the randomised patients had an aortic aneurysm, we assumed the EQ-5D score at any time point was the average of the EQ-5D at baseline for AAA patients presenting for elective repair (EVAR 1 trial), i.e. that their EQ-5D scores were 0.75, 0.74 and 0.74 at 3, 12 and 36 months, respectively (2).

Currency conversions

These used the PPP exchange rate (3).

Causal Analysis

A causal analysis in the ruptured AAA population only was conducted to address the question of what the effect of an endovascular strategy versus open repair in this population would be if everyone had adhered to the IMPROVE policy design (that is a CT scan plus EVAR if found anatomically suitable versus open repair). The causal analysis estimates the effect of the policy design in patients as randomised in a complier population (4). This provides an unbiased estimate of the true treatment effect, subject to certain modelling assumptions (5-7), which would not be possible with a per-protocol analysis, particularly when non-compliance is not at random.

Patients who were randomised to the endovascular strategy, found to be not anatomically suitable, based on evaluation at the local centre, and underwent open repair were classified as having complied with randomisation. Any other reasons for not receiving the allocated treatment were classified as non-compliance. Patients who died before repair were excluded from the analysis under the assumption that their outcome would be the same no matter what group they were randomised to. Patients who had a converted operation (EVAR converted to open) in the arm randomised to the EVAR strategy who were

anatomically suitable were treated as having adhered. Patients who had a converted operation after randomisation to open repair, were treated as having not adhered.

Categorisation of indications for e-interventions

These are based on the scoring system developed for elective AAA repair by the EVAR 1 trial investigators (8), with additional re-interventions, including those more common after emergency repair and for laparotomy-related complications) scored by IMPROVE trial investigators.

References

1. White IR, Royston P, Wood AM. Multiple imputation using chained equations: Issues and guidance for practice. Stat Med. 2011;30:377-99.

2 Brown LC, Powell JT, Thompson SG, Epstein DM, Sculpher MJ, Greenhalgh RM. The UK EndoVascular Aneurysm Repair (EVAR) trials: randomised trials of EVAR versus standard therapy. Health Technol Assess. 2012;16(9):1-218.

3 OECD. Purchasing Power Parities and Exchange Rates for all OECD countries. Organisation for Economic and Cooperation and Development, Paris, France. 2016 http://stats.oecd.org/Index.aspx?DataSetCode=PPPGDP. Accessed 24th February 2017

4. Cuzick J, Edwards R, Segnan N. Adjusting for non-compliance and contamination in randomized clinical trials. Stat Med. 1997;16:1017-29.

5 Bellamy SL, Lin JY, Ten **Have** TR.An introduction to causal modeling in **clinical trials.** Clin Trials. 2007;4(1):58-73

6 Sussman JB, Hayward RA. An IV method for the RCT: using instrumental variables to adjust for treatment contamination in randomised controlled trials. BMJ 2010;340:c2073

7 Díaz-Ordaz, KA; Franchini, A; Grieve, R; (2017) Methods for estimating complier-average causal effects for cost-effectiveness analysis.J Roy Statl Soc Series A. DOI: 10.1111/rssa.12294 (In Press).

8 Patel R, Sweeting MJ, Powell JT, Greenhalgh RM, EVAR trial investigators. Endovascular versus open repair of abdominal aortic aneurysm in 15-years' follow-up of the UK endovascular aneurysm repair trial 1 (EVAR trial 1): a randomised controlled trial. Lancet. 2016;388(10058):2366-74.

9 Davies L, Brown TJ, Haynes S, Payne K, Elliott RA, McCollum C. Cost-effectiveness of cell salvage and alternative methods of minimising perioperative allogeneic blood transfusion: a systematic review and economic model. Health Technol Assess. 2006;10(44):iii-iv, ix-x, 1-210.11

11 Dixon S, Mason S, Knowles E, Colwell B, Wardrope J, Snooks H, et al. Is it cost effective to introduce paramedic practitioners for older people to the ambulance service? Results of a cluster randomised controlled trial. Emerg Med J. 2009;26(6):446-51.

12 Curtis L. Unit costs of health and social care: University of Kent, UK (Personal Social Services Research Unit). 2012.
