# Supplementary material for: Comparative clinical effectiveness and cost effectiveness of endovascular strategy v open repair for ruptured abdominal aortic aneurysm: three year results of the IMPROVE randomised trial
Source: BMJ. 2017 Nov 14;359:j4859. doi: 10.1136/bmj.j4859 (PMC5682594; doi:10.1136/bmj.j4859)
Supplement: Supplementary file 3 — Appendix 3: Supplementary figures A and B [file pulu039133.ww3.pdf]

Appendix 3: Supplementary figures [posted as supplied by author]

Figure A Hazard ratios for overall mortality of endovascular strategy versus open repair for pre-specified subgroups (unadjusted analysis) in the full trial cohort.

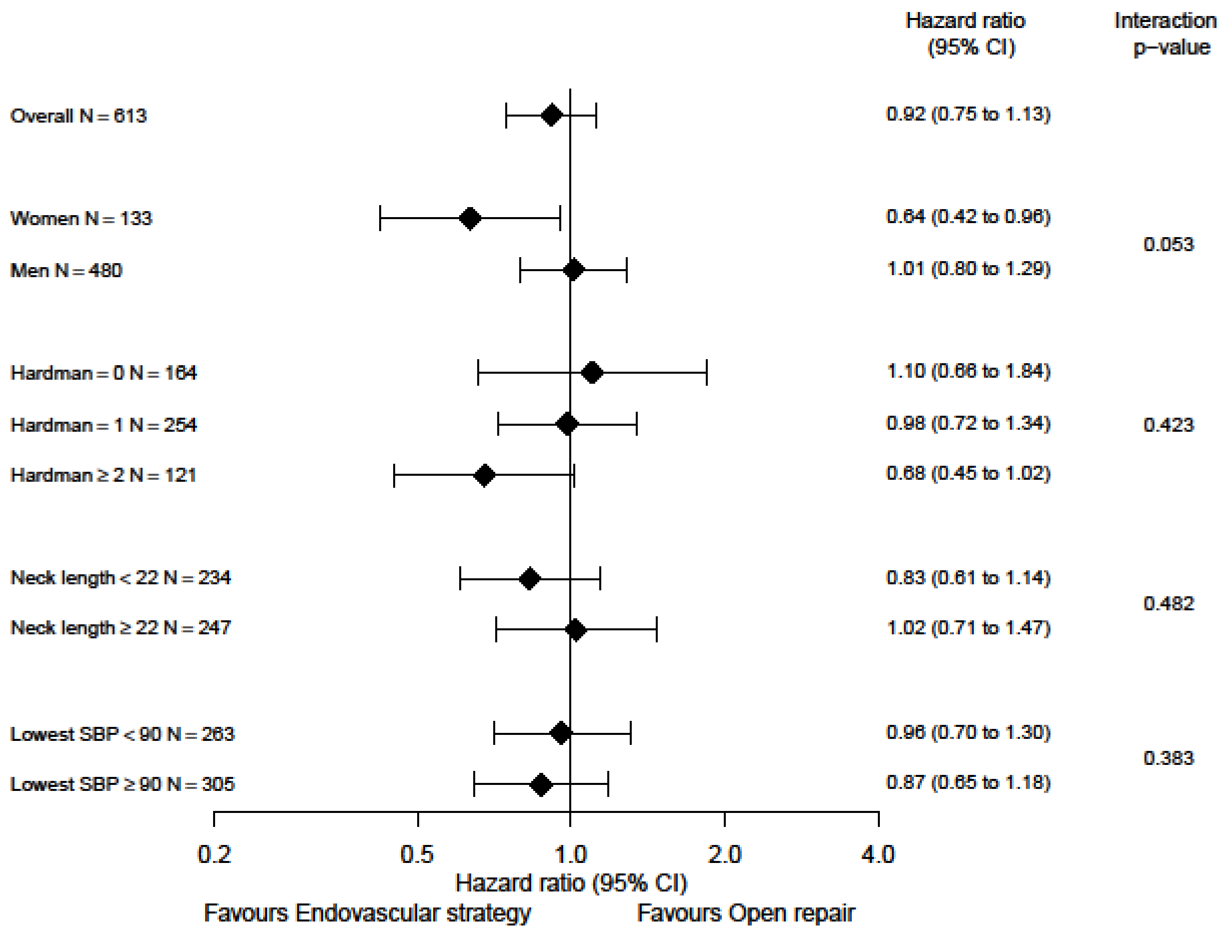

Figure B: Sensitivity analysis that considers the effect on the Incremental Net Monetary Benefit (at £30 000 per QALY) of alternative assumptions, compared to the base case for the full trial cohort (n=613).

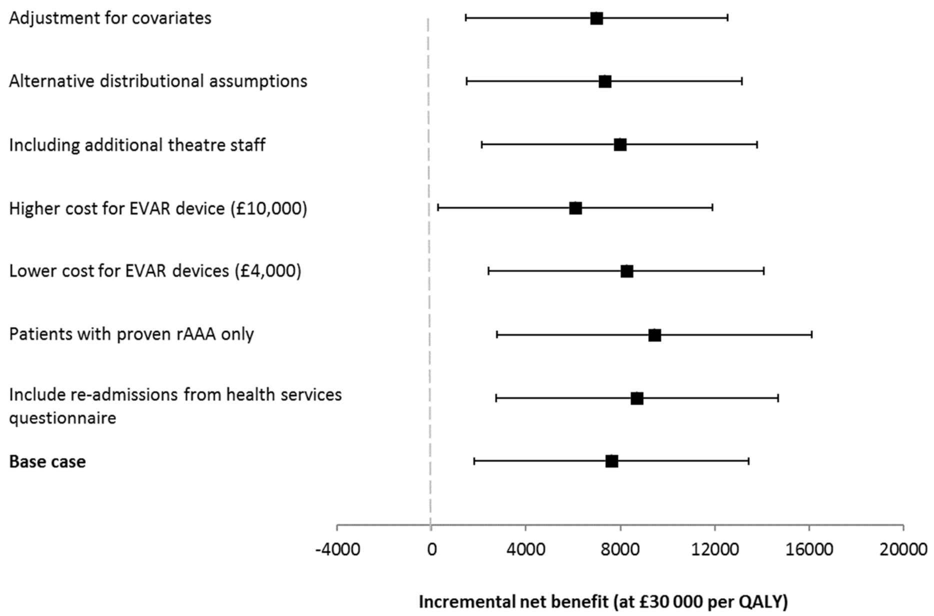

Notes: a) covariate adjustment was consistent with that for clinical outcomes. b) Gamma model (instead of Gaussian) was considered for skewed costs and QALYs. c) additional theatre staff (e.g. an additional radiologist and runner in the open repair arm) to reflect observed variation in theatre staff across trial centres were considered d) different costs for EVAR devices to reflect discounts obtained by some trial hospitals were considered. e) we reported cost-effectiveness inferences for patients with proven rupture. f) a conservative analysis that includes all hospital readmissions, including those for an intervention not related to the AAA or its treatment was considered.
